# Elimination of onchocerciasis in Africa by 2025: an ambitious target requires ambitious interventions

**DOI:** 10.1186/s40249-019-0593-x

**Published:** 2019-10-03

**Authors:** Robert Colebunders, Wilma A. Stolk, Joseph Nelson Siewe Fodjo, Charles D. Mackenzie, Adrian Hopkins

**Affiliations:** 1 Global health Institute, University of Antwerp, Antwerp, Belgium; 2 Department of Public Health, Erasmus MC, University Medical Center Rotterdam, Rotterdam, The Netherlands; 3 Task force for Global Health, Decatur, Georgia USA; 4 Neglected and Disabling Diseases of Poverty Consultant, Gravesend, Kent, UK

**Keywords:** Onchocerciasis, Elimination, Target, Morbidity, Epilepsy, Ivermectin

## Abstract

To achieve the elimination of onchocerciasis transmission in all African countries will entail enormous challenges, as has been highlighted by the active discussion around onchocerciasis intervention strategies and evaluation procedures in this journal.

Serological thresholds for onchocerciasis elimination, adapted for the African setting, need to be established. The Onchocerciasis Technical Advisory Subgroup of the World Health Organization is currently developing improved guidelines to allow country elimination committees to make evidence-based decisions. Importantly, onchocerciasis-related morbidity should not be forgotten when debating elimination prospects. A morbidity management and disease prevention (MMDP) strategy similar to that for lymphatic filariasis will need to be developed. This will require collaboration between the onchocerciasis elimination program, the community and other partners including primary health and mental health programs**.**

In order to reach the goal of onchocerciasis elimination in most African countries by 2025, we should prioritize community participation and advocate for tailored interventions which are scientifically proven to be effective, but currently considered to be too expensive.

## Multilingual abstracts

Please see Additional file 1 for translations of the abstract into the five official working languages of the United Nations.

## Background

On April 9th 2019, the World Health Organization (WHO) launched a global consultation for the 2021–2030 roadmap on neglected tropical diseases [1]. An important item on this new roadmap is the elimination of onchocerciasis in most endemic countries. Thanks to the efforts of the Onchocerciasis Control Programme in West Africa (OCP) and the African Programme for Onchocerciasis Control (APOC), during the last 30–40 years great progress has been made towards elimination of onchocerciasis as a public health problem in many African foci [2]. However to reach the elimination of transmission in all African countries will entail enormous challenges, as has been highlighted by the active discussion around onchocerciasis intervention strategies and evaluation procedures in this journal [3–6].

## Main text

Dadzie et al. in an opinion paper put the question: “Is onchocerciasis elimination in Africa feasible by 2025” in perspective, based on lessons learnt from both OCP and APOC [3]. They recognise the success of the Onchocerciasis Elimination Program for the Americas in eliminating onchocerciasis transmission in 4 of 6 endemic countries in South America, but consider that the adoption of serology-based criteria for elimination, as used in South America, has unnecessarily prolonged interventions in many areas where APOC’s own entomological and parasitological criteria for elimination [2] were possibly met already.

In response, Cupp et al. [4] and Richards et al. [5] maintain that the American experience in the fight against onchocerciasis provides a wealth of research and technical know-how that have already, and would continue to benefit the African continent. They highlight the strategies of twice or more rounds of mass drug administration (MDA) of ivermectin per year, as well as the use of OV16 serology as a decision tool to stop MDA.

These authors disagree about the appropriateness of serology-based criteria for evaluating onchocerciasis elimination in Africa. Indeed, the comparative validity of parasitological versus serological criteria for stopping MDA is unknown. WHO stepped away from using skin snip-based parasitological criteria, in view of its reduced sensitivity at low parasite levels and its invasiveness. Yet, the evidence underlying the more recent serological criteria is still rather weak and optimal ELISA protocols still need to be defined [7]. The WHO Onchocerciasis Technical Advisory Subgroup (OTS) is in fact working on the evidence base in order to identify strategies to help country elimination committees make valid decisions. Evidence-based serological thresholds, adapted for the African continent, would need to be established. Monitoring for resurgence of onchocerciasis transmission will equally be required. The development of a more sensitive antibody test by integrating multiple biomarkers *of Onchocerca volvulus* infection may lead to a reduction in the number of children to test when assessing elimination thresholds [8].

The contributors to the current discussion all agree that intensified efforts are needed to achieve the ambitious elimination goal, at least in some areas. APOC had aimed to achieve elimination in 80% of African countries by 2025 [9], but today it is unlikely that this target will be reached with the current onchocerciasis elimination strategies and available funding. Onchocerciasis-endemic countries in both Africa and South America show considerable variation in the characteristics of the disease and transmission dynamics. Therefore interventions need to be tailored to each onchocerciasis focus.

In hyper-endemic areas with high onchocerciasis-associated morbidity like onchocerciasis-associated epilepsy (OAE), aggressive strategies such as 6-monthly MDA with high coverage and complementary vector control should be deployed, as was the case in northern Uganda [10]. Such interventions are often considered too expensive, but may turn out to be cost-effective by decreasing morbidity and mortality. Moreover, onchocerciasis morbidity is often the driving factor that will increase community participation and therefore coverage and ultimate success. In hypo-endemic areas, annual community-directed treatment with ivermectin (CDTI) may suffice to stop transmission within 6–8 years, but emphasis must be laid on achieving ≥85% coverage of eligible population. The need for tailored interventions underscores the need for more information, for wider thinking and continuing investigation into the various components of this disease complex (e.g. new assessment tests, better understanding of the clinical disease, reassessing the chemotherapeutic regimes, understanding the clinical and transmission significance of hypo-endemic areas).

In this debate about onchocerciasis elimination, the elimination of onchocerciasis-related morbidity should not be forgotten. It has been suggested that onchocerciasis is not a public health problem anymore [1, 3]. This is evident for many regions, but is definitely not true throughout Africa [11]. Recent studies highlighted OAE as a major unrecognised public health problem in many remote onchocerciasis foci where there is inadequate ivermectin coverage such as in parts of the Democratic Republic of Congo [12], Cameroon [13], Tanzania [14] and South Sudan [15]. This also applies to onchodermatitis, which still exists in many endemic locations. Assessments of the clinical disease are rarely done in national onchocerciasis programs [16]. Onchocerciasis elimination programs in Africa should take into account OAE and the other clinical presentations of this infection in their elimination and surveillance strategies, and a morbidity management and disease prevention (MMDP) strategy similar to that for lymphatic filariasis will need to be developed [17, 18]. This will require collaboration between the onchocerciasis elimination program with other partners including primary health and mental health programs**.**

## Conclusions

In developing the roadmap towards onchocerciasis elimination, decision-makers should strive to implement the most effective strategies (bi-annual CDTI, vector control, etc.) albeit their relatively higher costs. As was so successfully done for human immunodeficiency virus infection, the person living with the infection should be the focus of our efforts, not the parasite and not the available budget. It is important to involve the affected communities and advocate for tailored, evidence-based interventions. Finally, we should keep the 2025 target for stopping treatment, but clearly a paradigm shift will be needed.

## Supplementary information

**Additional file 1:** Multilingual abstracts in the five official working languages of the United Nations. (PDF 298 kb)

## Data Availability

Not applicable.

## Abbreviation

APOC: African Programme for Onchocerciasis Control
CDTI: Community-directed treatment with ivermectin
MDA: Mass drug administration
OAE: Onchocerciasis-associated epilepsy
OCP: Onchocerciasis Control Programme in West Africa
WHO: World Health Organization
