# Supplementary material for: Elimination of onchocerciasis in Africa by 2025: an ambitious target requires ambitious interventions
Source: Infect Dis Poverty. 2019 Oct 3;8:83. doi: 10.1186/s40249-019-0593-x (PMC6775645; doi:10.1186/s40249-019-0593-x)
Supplement: Supplementary file 1 — Additional file 1: Multilingual abstracts in the five official working languages of the United Nations. (PDF 298 kb) [file 40249_2019_593_MOESM1_ESM.pdf]

Translation of the abstract into the five official working languages of the United Nations

القضاء على داء كلابية الذنب في أفريقيا بحلول عام 2025: الهدف الطموح يتطلب تدخلات طموحة

كوليبندرز روبرت، ويلما أ. ستولك، سيوي فودجو جوزف نيلسون، شارلز د. ماكنزي، هوبكنز أدريان

موجز
إن تحقيق القضاء على انتقال داء كلابية الذنب في جميع البلدان الأفريقية يترتب عليه تحديات هائلة، هذا ما تؤكد عليه المناقشة النشطة حول استراتيجيات التدخل وإجراءات التقييم بخصوص داء كلابية الذنب في هذه النشرة.
يجب تحديد عتبات مصلية للقضاء على داء كلابية الذنب، تكون ملائمة للبيئة الإفريقية. تعمل المجموعة الفرعية الإستشارية التقنية لداء كلابية الذنب التابعة لمنظمة الصحة العالمية WHO في الوقت الحالي على تطوير مبادئ توجيهية محسّنة للسماح للجان خاصة بالقضاء على داء كلابية الذنب في البلد باتخاذ قرارات تستند إلى أدلة. والأهم من ذلك، أنه لا ينبغي نسيان الإعتلالات المرضية المرتبطة بداء كلابية الذنب عند مناقشة آفاق القضاء عليه. سيكون هناك حاجة إلى تطوير استراتيجية لمعالجة الإعتلالات المرضية والوقاية من الأمراض (MMDP) مماثلة لاستراتيجية داء الخيطيات اللمفاوية. سيتطلب ذلك تعاوناً بين برنامج القضاء على داء كلابية الذنب، والمجتمع وشركاء آخرين بما في ذلك برامج الصحة الأولية والصحة العقلية .
من أجل الوصول إلى هدف القضاء على داء كلابية الذنب في معظم البلدان الأفريقية بحلول عام 2025، ينبغي أن نعطي الأولوية لمشاركة المجتمع والدعوة إلى صياغة تدخلات ثبت علمياً أنها فعالة، ولكنها تعتبرحالياً مكلفة للغاية .

Translated from English version into Arabic by Ammar Alhosain, Revised by Reem Zakkar, through

TRANSLATORS WITHOUT BORDERS

2025 年非洲消除盘尾丝虫病：远大目标需要强有力的干预措施

Colebunders Robert, Wilma A. Stolk, Siewe Fodjo Joseph Nelson, Charles D. Mackenzie, Hopkins Adrian

摘要
正如相关研究显示，在所有非洲国家实现盘尾丝虫病的阻断传播面临巨大的挑战。首先，需要结合非洲的情况，设定评价盘尾丝虫病消除的血清学阈值。目前，世界卫生组织盘尾丝虫病技术咨询小组正在更新指导方针，允许非洲国家消除委员会制定循证决策。在讨论消除前景时，也需要考虑到与盘尾丝虫相关疾病的发病率。例如，制定类似于淋巴丝虫病的发病率管理和疾病预防（MMDP）策略。这需要盘尾丝虫病消除项目、社区以及和包括初级保健和心理健康项目等其他机构之间的合作。为实现 2025 年大多数非洲国家消除盘尾丝虫病的目标，虽然现行干预措施已证实有效，但成本较高，我们应优先鼓励社区参与，并倡导精准干预。

Translated from English version into Chinese by Cong-Shan Liu, edited by Jin Chen,

CHINESE CENTER FOR DISEASE CONTROL AND PREVENTION
NATIONAL INSTITUTE OF PARASITIC DISEASES

Élimination de l'onchocercose en Afrique d'ici 2025: un objectif ambitieux nécessite des interventions ambitieuses

Colebunders Robert, Wilma A. Stolk, Siewe Fodjo Joseph Nelson, Charles D. Mackenzie, Hopkins Adrian

Résumé
L'élimination de la transmission de l'onchocercose dans tous les pays africains entraînera d'énormes défis, comme l'a souligné la discussion active sur les stratégies d'intervention et les procédures d'évaluation de l'onchocercose dans ce journal.
Des seuils sérologiques pour l'élimination de l'onchocercose, adaptés au contexte africain, doivent être établis Le sous-groupe consultatif technique de l'OMS(Organisation mondiale de la santé) sur l'onchocercose élabore actuellement des directives améliorées pour permettre aux comités d'élimination des pays de prendre des décisions fondées sur des preuves. Il est important de noter que la morbidité liée à l'onchocercose ne doit pas être oubliée lors des débats sur les perspectives d'élimination. Une stratégie de gestion de la morbidité et de prévention des maladies similaire à celle de la filariose lymphatique devra être élaborée. Cela nécessitera une collaboration entre le programme d'élimination de l'onchocercose, la communauté et d'autres partenaires,notamment des programmes de santé primaire et de santé mentale **.**
Afin d'atteindre l'objectif de l'élimination de l'onchocercose dans la plupart des pays africains d'ici à 2025, nous devons accorder la priorité à la participation de la communauté et plaider en faveur d'interventions sur mesure dont l'efficacité scientifique a été prouvée, mais qui sont actuellement considérées comme trop coûteuses.

Translated from English version into French by Aghilas Mihoub, Revised by Ahmad Dabaghzadeh, through

TRANSLATORS
WITHOUT BORDERS

Ликвидация случаев онхоцеркоза в Африке к 2025 г.: амбициозная задача требует широкого подхода

Colebunders Robert, Wilma A. Stolk, Siewe Fodjo Joseph Nelson, Charles D. Mackenzie, Hopkins Adrian

Аннотация
Достижение ликвидации передачи онхоцеркоза во всех африканских странах является чрезвычайно сложной задачей, о чем свидетельствует активное обсуждение стратегий и процедур оценки онхоцеркоза в этом журнале.

Необходимо установить серологические пороговые значения при ликвидации онхоцеркоза, специально адаптированные к африканским условиям. В настоящее время Техническая консультативная подгруппа по онхоцеркозу ВОЗ разрабатывает обновленные руководства, позволяющие страновым комитетам по элиминации принимать основанные на фактических данных решения.   Важно отметить, что при обсуждении перспектив элиминации не следует забывать о заболеваемости, связанной с онхоцеркозом. Необходимо разработать стратегию по контролю за заболеваемостью и профилактикой болезни, аналогичную таковой для лимфатического филяриатоза. Это потребует сотрудничества между программой ликвидации онхоцеркоза, сообществом и другими партнерами, включая первичное звено медицинской помощи и программы психического здоровья**.**

Чтобы достичь цели ликвидации онхоцеркоза в большинстве стран Африки к 2025 году, следует уделить приоритетное внимание участию общественности и выступать в поддержку специально подобранных мероприятий, которые с научной точки зрения доказали свою эффективность, но в настоящее время считаются слишком дорогостоящими.

Translated from English version into Russian by Veronika Demeshchyk, Revised by Anna Kukharchuk, through

TRANSLATORS
WITHOUT BORDERS

La eliminación de la oncocercosis, en África, antes de 2025:   un objetivo ambicioso requiere intervenciones ambiciosas.

Colebunders Robert, Wilma A. Stolk, Siewe Fodjo Joseph Nelson, Charles D. Mackenzie, Hopkins Adrian

Resumen

Lograr la eliminación de la transmisión de la oncocercosis, en todos los países africanos, implica enormes desafíos, como se ha puesto de relieve en el debate activo sobre las estrategias de intervención de oncocerciasis y los procedimientos de evaluación en esta revista.

Para eliminar la oncocercosis es necesario establecer umbrales serológicos adaptados al entorno africano. El Sub-grupo de Asesoramiento Técnico sobre Oncocercosis de la OMS está desarrollando directrices mejoradas para permitir que los comités de eliminación de los países tomen decisiones, con base en evidencia.   Es importante destacar que la morbilidad relacionada con la oncocercosis no debe olvidarse, al debatir las perspectivas de eliminación. Será necesario desarrollar una estrategia de gestión de la morbilidad y prevención de enfermedades (MMDP) similar a la de la filariasis linfática. Esto requerirá la colaboración entre el programa de eliminación de oncocercosis, la comunidad y otros socios, incluidos los programas de salud primaria y salud mental**.**

Con el fin de alcanzar el objetivo de la eliminación de la oncocercosis, en la mayoría de los países africanos para el año 2025, deberíamos dar prioridad a la participación de la comunidad y abogar

por intervenciones específicas, las cuales hayan demostrado ser científicamente eficaces; no obstante, actualmente se consideren demasiado caras.

Translated from English version into Spanish by María Luz Puerta, Revised by Rosanna Lenci, through

TRANSLATORS
WITHOUT BORDERS
